# Perceptions of provincial and district level managers’ on the policy implementation of school oral health in South Africa

**DOI:** 10.1186/s12913-020-06004-9

**Published:** 2021-01-06

**Authors:** Mpho Molete, Aimee Stewart, Aneesa Moolla, Jude Ofuzinim Igumbor

**Affiliations:** 1 School of Oral Health Sciences, Department of Community Dentistry, University of the Witwatersrand, Johannesburg, South Africa; 2 School of Therapeutic Sciences, University of the Witwatersrand, Johannesburg, South Africa; 3 School of Public Health, University of the Witwatersrand, Johannesburg, South Africa

**Keywords:** Policy implementation, Oral health, Governance, School health

## Abstract

**Background:**

Although school oral health programmes have been ongoing for years, there is little evidence to show how their policy elements are governed or translated into tangible implementation activities and population outcomes at the district level. The need for such a study is heightened by the persistent burden of oral health conditions and unmet oral treatment needs of South Africa’s children. This study therefore sought to describe provincial and district level managers’ perceptions of school oral health policy, and to identify gaps and conditions needed for successful policy implementation.

**Methods:**

This was an exploratory qualitative study where eight oral health managers from the Gauteng provincial and district offices were purposively sampled. Data were collected using interviews and a policy review rubric. The 10 Siddiqi governance principles framework was used to guide the data analysis.

**Results:**

The managers’ perceptions and the policy document review indicated that national policy covered the principles of strategic vision, responsiveness to health needs, equity and inclusivity with clarity; however these principles were not translated consistently by the managers at a local level. Policy gaps were identified in the areas of stakeholder involvement, accountability, reliable information systems and ethical guidelines. Much of the gaps in policy translation were attributed to inadequate human resources and poor communication processes by the national leadership to support district level implementation.

**Conclusions:**

There were inconsistencies in policy awareness and translation in the districts and hence an in-depth review of the policy translation gaps is paramount to its efficient resolution in the context of resource and capacity limitations. Furthermore, optimizing multi-sectoral participation and identifying shared, novel and practical solutions to policy translation impediments is necessary.

## Background

In South Africa, even though school oral health programmes have been ongoing for over 20 years, the services provided at the schools are reported to be inconsistent and fragmented [1–3]. This is in spite of the widely referenced national and provincial evidence based recommendations contained in the oral health strategic policy documents [2, 4]. South African studies report that some schools are offered once-off activities and others are serviced only once a year [1–3]. Upon assessing the gaps between policy and practice from the perspectives of frontline oral hygienists implementing the school oral health programmes, Molete et al., [3] observed the behavior of frontline oral hygienists as they traded policy direction with poor quality interventions in response to the structural challenges to policy implementation.

The main policy document guiding oral health in South Africa is the National Oral Health Strategy [5]. The national strategy provides an oral health framework for provincial and district governments who are then tasked with translating it into operational plans for frontline implementation, thus all the operational documents are informed by the national oral health strategy. One of these is the Gauteng Provincial Oral Health Strategy [6]. The provincial strategic document recognizes the national goal of reducing the burden of oral diseases by 2030 in its strategic approach to prioritize child oral healthcare access through school based programmes; increase the percentage of 6 year olds who are caries free to 60%; and to reduce 12 year olds Decayed Missing Filled Score (DMFT) score to an average of 1.0 [6].

Gauteng Province of South Africa has an oral health head office which is responsible for leading provincial and district oral health services. The provincial oral health services are offered through three university based dental health facilities which are responsible for primary, secondary and tertiary services. The districts’ facilities offer primary health services, some limited secondary services and are located across five health districts within the Province of Gauteng [5]. Each district has a dental manager that oversees all the districts’ operational needs. In addition to a dental manager, each district also has an oral hygiene manager who is responsible for the oral hygiene work which includes preventative care and community oral health services [5, 6].

The dental and oral hygiene district managers are responsible for coordinating the attainment of national and provincial strategic objectives at a local level [7]. Consequently, the district managers are responsible for how policy is experienced by oral healthcare users and have the discretionary power to decide what actions are appropriate and what problems should be prioritized for action in the context of resource limitation and the local disease burden [8, 9]. Therefore, they have a key role to play in how policy implementation unfolds at a local level. Their judgements are affected by their values and beliefs, such as personal experiences, education and training, organizational norms and cultural norms [7]. Their values and beliefs are translated to core policy positions that influence their response to policy proposals and how they decide to engage with a particular policy [9]. Hence it is necessary to get an understanding of how they operate and how their actions influence policy implementation.

Policy implementation processes are commonly categorized as top-down or bottom-up [9, 10]. A top-down approach is often formulated at a national level and then translated to operational instructions that are used by implementing officials at a local community level. The process is rigid and tends to undermine the involvement of local implementers who are expected to carry out the work [9]. The bottom-up approach is iterative as incremental steps of change and revisions are made between local implementers and national leaders [9]. This approach also emphasizes the role of local implementers in the implementation process in terms of the discretionary authority they have in carrying out the policy within a local context [8].

The two approaches both have their limitations however they complement each other in navigating through the complexities of policy implementation [10]. Hence the development of hybrid approaches that combine top-down mechanisms to promote good practice together with bottom-up persuasive orientated mechanisms [11]. Central to the hybrid integrated approaches are the relationships between and within organizations for managing deliverables, risks and promoting collaborations [12]. For good governance all key stakeholders need to be represented to ensure transparency, enhance understanding of decisions made, as well as share the strategic and operational processes [13]. Poor governance processes and lack of engagements often lead to systems with dysfunctional cultures and lack of effective leadership [13].

A major component of policy implementation is about governing actors and organizations working together towards attaining policy objectives [14]. Hence governance is key for overall health systems development and understanding governance is important in improving health systems performance [15, 16]. The Siddiqi governance assessment framework will therefore be used in this study as it is broad and integrates questions that encompass governance principles from national policy formulation to policy implementation [16]. The use of this framework is aimed at analyzing the policy response of oral health managers at provincial and district levels in driving the implementation of school oral health services. This framework additionally assists in outlining the policy gaps and conditions needed for successful implementation. Please see Table 1 for a description of the 10 key principles ranging from strategic vision to the ethics of policy implementation.

**Table 1:** Governance Framework Principles

Health Systems Governance Principles	Description
Strategic vision	There needs to be clear understanding of policy objectives and long term vision
Participation and consensus orientation	Participation is necessary among all stakeholders and there should be clarity on how decisions are finalized.
Equity & inclusiveness	The allocation of public sector resources needs to be equitable and address financial barriers of the poor.
Rule of law	Policies need to be translated to laws or rules which need to be enforced.
Transparency	There needs to be transparency in decision making and allocation of resources.
Responsiveness to patient health needs	Policies need to be responsive to the needs of the population.
Effectiveness and efficiency	The human resource systems and communication processes from leadership need to be effective.
Accountability	Mechanisms for overseeing adherence to financial, administrative rules need to be in place.
Intelligence and information	There needs to be adequate health systems information and it should be accessible and reliable so that it can be used for decision making.
Ethics	Importance needs to be attached to ethics in research and services.

Source: Adopted from Siddiqi et al. [16]

## Methods

This is an exploratory qualitative study as there is little pre-existing knowledge on managers’ perspectives on policy implementation of oral health programmes [17]. This method provides an opportunity to explore the topic in-depth in order to understand the context and the meaning behind the provincial and district managers’ perspectives in terms of policy implementation [18].

The study population consisted of oral health managers from the Gauteng districts and provincial offices. There are a total of 12 oral health managers in the Gauteng province, and a purposive sample of eight managers responsible for school oral health were selected from the following districts Tshwane, Central Johannesburg, West Rand, Erkhululeni, Sedibeng and West Rand and two from the Gauteng provincial head office. Of the eight participants; two were dentists, one dental therapist and five were oral hygienists. These key-informants were the most appropriate in providing access to the information that was needed as they are responsible for operational planning and supervision of the oral hygienists who were implementing school oral health services [19].

Data were collected through in-depth interviews and policy document review. The semi-structured interview guide consisted of questions concerning: policy, programme objectives, school health services and education, school and community relationships and nutritional services [20]. The policy documents reviewed included the National Oral Health Strategy and the Gauteng Oral Health Strategy, [5, 6]. In addition, internal operational plans that included the Community Oral Health Service Manual and Gauteng Oral Health Promotion Strategy were also reviewed. The interview guide was used in managing the questioning and allowed the interviewer the flexibility of probing according to the participants’ responses [21]. The first author conducted the interviews at the offices of the participants; the interviews were approximately an hour long, and were audio recorded and transcribed verbatim [18]. Field notes were additionally compiled after each interview to gather information on the context of the setting.

### Data analysis

A framework analysis was utilised following the steps outlined by Ritchie et al. [18]. The framework analysis included familiarisation, identifying a thematic framework, indexing, charting and finally, mapping and interpretation [18]. The analysis was iterative and the first and fourth authors reviewed the transcripts and policy documents multiple times for familiarisation. Identifying a thematic framework involved a generation of initial codes deductively and aligned to the conceptual framework; this was done by the first author. The first and fourth authors further identified other emerging codes inductively to build the thematic framework. This was done until the data were saturated. Indexing involved aligning the selected codes to the constructs of the conceptual framework. Specific themes were arranged according to charts on a matrix to ensure that they reflected the descriptions of the participant and were aligned to the conceptual framework constructs. The findings of the policy analysis were also charted in alignment with the conceptual framework and were used to support the statements made by the participants. All the authors reviewed the charts to check for potential biases. This then further guided the mapping and interpretation of the data as the themes were reviewed and refined [21].

The four criteria of trustworthiness which include credibility, dependability, transferability and confirmability were addressed [22]. In terms of credibility, all authors reviewed the data and addressed biases in order to ensure that the phenomenon was accurately described. The third author then re-coded the data, cross checked the entire process and resolved discrepancies in order to improve dependability. In addressing transferability, the context of the field work was well described so an assessment could be made on whether a similar method can be applied to another setting. Finally, confirmability was ensured by utilizing field notes for making analytical sense of the data and additionally, data were shared with some of the participants in order to confirm that the data were truly represented [22].

## Results

As outlined in Table 2, the results discussed reflect the findings of both the policy document review and the perspectives of the provincial-district managers.

**Table 2:**
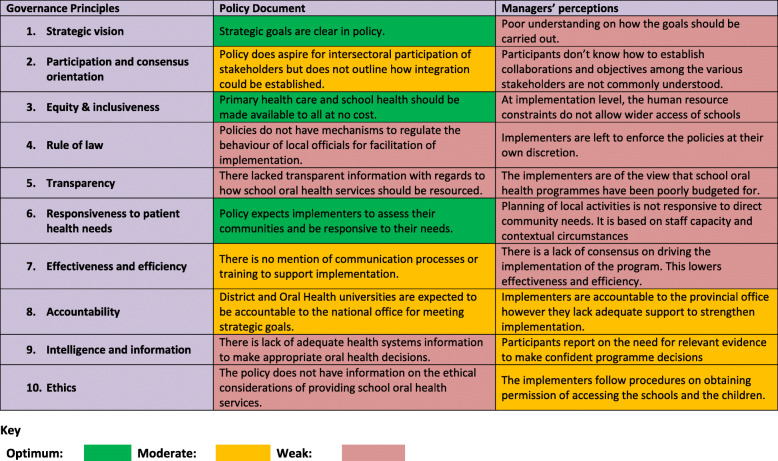
Assessment of Governance Principles

### Strategic vision

Although the national strategy does not have a vision statement, its key goals are clear and designed to improve oral health services for all citizens. The goals are to be attained as follows; 60% of 6–7 year olds to have fissure sealants placed on first permanent molars; 40% of 11–12 year olds to have fissure sealants placed on second molars and 50% of primary school children in the country to be exposed to a tooth-brushing programme. Furthermore, 20% of primary schools in each province to be on an oral health programme each year. However, it appears that activity targets on how to achieve the main goals are poorly understood by the oral health managers and there is no clarity on how each district aims to achieve their targets. This point is illustrated by these excerpts from the managers interviewed:

*“Well, we have a target from national, the target from national is about 50 schools for Gauteng, but they try by all means to reach as many as possible, because I remember the other year they were sitting at 520 schools (For toothbrushing programmes).”* ***P1***

“*We’re focusing on Grade R, 1, 2 and 3. Uhhh, it is crazy, you know inside those schools there are 600 children in the Grade 1, 2 and 3. Then maybe they would go to another school and also accommodate the Grades R, 1 and 2 there, it all depends, it depends on Oral hygienists, Oral hygienists by now they know their areas…they know the schools”.* ***P3***

### Participation and consensus orientation

The national and provincial strategies briefly outline the need for communication links and regular annual meetings between national and provincial offices; there is no mention of participation from district leaders, interdisciplinary departments and the community. Integration of oral health into other health programmes, and co-ordination of stakeholder collaboration has also been documented in the policy. However, none of the documents mention how the collaborations and integrated decision making could be established. There is an integrated school health policy developed by the National Department of Basic Education and Health, however there appears to be no written co-operative guideline between local departments of health and education in carrying out the school health policy. In this regard, one of the participating managers remarked that:

*“I mean the years that I’ve been working here and then when you go to the meetings, it’s always said that there’s an agreement between department of health and department of education, but when you go down to the level of the schools, nobody knows about this agreement “. P6*

Inter-organisation collaboration is perceived negatively and one of the managers suggested that it may possibly be effective at top management as it has not translated adequately at an operational level. This is attributed to the challenges of large classes and inadequate resources that the managers experience.

*“They (referring to executive management) think, it’s a good thing , because if your manager is sitting in an office, obviously, yes...you know, for them it’s an excellent concept, but if you’re operational, it is completely different”.****P5***

### Equity and inclusiveness

The national strategy is founded on the human rights articulated by the constitution of South Africa in terms of access to health care irrespective of gender, age, ethnicity, culture, religion or geographic location. A comprehensive primary healthcare service package in oral health is therefore available for all individuals irrespective of financial standing. Thus all district primary health care services are free, including school oral health services. This addresses equity and inclusiveness. However according to the oral hygienists, the human resource limitations faced by school oral health hinder the delivery of screening and services to the wider school population. Another participant added that:

*“They are not expected to screen everybody at the schools because like I indicated, in terms of the human resource, we’re short, understaffed, in terms of Oral Hygienists”.* ***P1***

### Rule of law

The strategic and operational documents reviewed do not have mechanisms or incentives alongside their recommendations to regulate the behaviour of local officials in order to facilitate effective implementation. Districts are expected by the national office to customise their interventions according to the specific needs of a community and the available resources. However, the managers do not work according to a systematic plan for customising their interventions. Instead they allow the implementing hygienist to use their discretion in customizing activities; this then subsequently translates to coping mechanisms, such as selecting smaller schools or classes that were cooperative, with the plan of making working life easier and reaching performance targets successfully. The participant’s views are expressed in the quotes:

*“No, we’re not prescriptive. Each district has their own way of working”* ***P1***

*“We, we’ve sort of formulated a criteria in terms of fissure sealants and to choose the smaller schools because in the future we find it doesn’t help for us to go to a big school. We’re only 2, so, it’s better to choose smaller schools so that you start the programme and then you cover all the children”* ***P6****.*

### Transparency

The provincial strategy and local operational policies lack transparent information with regards to how school oral health services should be resourced in terms of staff and equipment. Oral health finances are integrated within the district budgets and there is no ring-fenced budget for specific oral health activities, thus oral health programmes are expected to request resources from the province and district at their point of need. The system appears to constrain the local managers in acquiring adequate resources and lack clarity on the level of resources available for their programmes. They therefore perceive resources for school oral health to be inadequately allocated as show by the remarks:

*“They have posts for dentists but the posts for oral hygienists and dental therapists are limited, that’s the group, that we should be targeting because we want to prevent..”****P5***

This limitation impacts on widening the reach of school children and on what the oral hygienists are able to do at the schools.

*“I can’t really say it’s according to the need because everybody needs, all the school’s needs but I only have 5 oral hygienists. So, they are concentrating on certain schools or whatever they have time for.”****P3***

### Responsiveness to patient health needs

The national and provincial strategies expect the districts to assess the oral health condition of the community, prioritise needs, identify resources available and select appropriate activities. The participants however added that there is a lack of consistency in response to the question on how to plan for their activities as they get to the different schools:

“*Oh, ok, remember they have to screen the children first. So from the screening they will be able to determine what type of treatment, will be .”* ***P1***

“*Ok, they first must try to offer the brushing programme ok? it’s policy and obviously they are linked to targets”.* ***P4***

“*With, with us in Gauteng , we’ve got a community manual that advises us on what we do and when”.* ***P2***

It is also apparent that some of them follow guidance from internal procedural documents that have been compiled over the years by the hygiene managers. This observation is demonstrated in quotation:

*“The oral hygienists also refer to a community oral health manual; therefore there are few other internal working documents they are referring to. You know, we have a really nice manual. We have a Community Services, Community Oral Health Services manual.*

The internal documents mentioned include the Community Oral Health Service Manual and the Gauteng Oral Health Promotion Strategy document. The Community Oral Health Services manual, summarizes the goals of the national and provincial strategies, and includes logistical operational procedures for school activities. It provides information on how the oral hygienists should assess the school community; examine the oral health needs of the children, the equipment they should take and the administrative documents they should complete. The Oral Health Promotion Strategy is very similar to the Community Oral Health Service manual; the only difference is that it does not include the operational procedures. The two internal documents are however not official, they are not dated and thus there is no indication on when they were drafted or disseminated.

### Effectiveness and efficiency

The national strategy does have a monitoring and evaluation plan and has included tools that the managers can utilise for assessing their communities and reporting appropriately. However, it does not mention any plans for training or capacity development of provincial or district implementers in using the tools. Hence, at an operational level where the districts are expected to formulate direction in achieving the goals, there is no evidence that the monitoring and evaluation tools are being utilised and consensus is lacking. This view is illustrated by the quote from one of the participants:

*“In the district, we’ve been trying to find innovative ways to do...to carry out these programmes but it’s we’re at a standstill. We don’t know, we don’t know.”****P6***

The managers have therefore attempted to set targets that are aimed at not only achieving the broad national goals but are also used for monitoring work performance. These targets however differ per district and there are inconsistencies on how they are set and interpreted. This has caused some discontent among implementing oral hygienists.

*“So, that’s (referring to targets) also a confusing thing for our hygienists. The one says this, the other one says that...and you know what, that the districts all seem to be doing, what they want. You know what, if you are in your province, Gauteng province, you need to stick to the same thing”* ***P4***

### Accountability

The national strategy outlines how all the district and provincial services should be accountable to the national office at meeting the strategic goals. The National Department is responsible for leading the processes of formulating, implementation and reviewing the oral health strategy. In addition, it is to establish norms and standards for oral health service delivery as aligned to existing policy. The National Director for oral health collaborates with the academic oral health leads from universities that have dental schools in matters concerning policies for oral health services in the country. The provincial departments are expected to guide the district services in customising the interventions and setting operational targets; the structure follows a hierarchical top down approach where policy is centrally controlled.

Although the oral health managers interviewed indicated that they are given the opportunity of proposing policy direction changes, they have to follow the system of command and can only approach the National Office when they have evidence based information from the academic sector.

*“Management decision can change how things are done, particularly when programme is not working, they could change direction, provided the National Office agrees and the universities provide evidence”.* ***P1***

The managers reported that it is not always easy to get academic support when they want to change policy direction; responsiveness from some of the academics is not as forthcoming as they would like it to be.

*“It’s a sore point. For me personally, I sometimes or most of the times feel that academic world is very far removed from the real world ok, I get very upset when I look at the number of academics doing researches, extensive research and I look at what is happening practically, it’s like we’re worlds apart.”****P5***

### Intelligence and information

This refers to the availability of adequate health systems information for making appropriate oral health decisions. This was not reflected in the national and provincial strategy documents as the oral health epidemiological information is that of 2002 and thus not useful for making current decisions and ongoing appropriate oral health planning. Although there are pockets of recently published regional epidemiological information, none of them are reported in the strategic documents and furthermore they do not adequately reflect the oral health needs of the wider population. This results in the managers’ lack of confidence in making concrete decisions as expressed in remarks of the interviewees:

*“Because we know it’s good to brush teeth and we know it’s good to protect and to put fissure sealant, but we don’t know out there in the community, we actually don’t have the proof, you know the evidence base that shows its working in the schools”.* ***P6***

They indicate that they would like university academics to share their expertise in evidence based practice so that they could make better decisions.

*“Well, in terms of reporting, we will see data that they are doing according to the goals and their job descriptions, but I still feel if we can get an engagement and support from the universities in terms of research, to find out, are we really making an impact because, one can come and say, I’m doing so many restorations, so many fissures but, was it a quality treatment, is it indeed helping, are we wining?”****P1***

### Ethics

The national strategy does not include the importance of aligning ethical principles in research and oral health services. However at an operational level the oral hygienists follow the appropriate ethical procedures for accessing schools and children at the schools.

*“So, as department of health, the role that we play, we engage with the department of education so that we can be given permission to access the children in the schools, and so that were able to screen them and identify the, problems that they may have in terms of oral health and then, start to plan on how to go about treating the children”* **P1**

## Discussion

Our study shows that although the policy goals appears to be clear on paper, there is a lack of clarity at an operational level on what the key policy objectives are and how to achieve them. The policy aspirations of intersectoral collaboration were not realised as there was no clear framework at a national level. There was no policy direction on law enforcement and transparency with resource allocation. The principles of responsiveness and equity were not fully translated to policy action due to poor interpretation of how they were meant to be carried out. Much of the policy gaps were attributed to poor communication processes by the national leadership to support local implementation. Further, the policy lacked information on accountability, reliable information systems and ethical guidelines. This left the implementers to utilise their discretion in devising their own strategies which were uncoordinated.

The national oral health policy in South Africa is largely derived from a top-down approach as it was developed by the leadership at a national level. Although the managers attempted to operationalise the policy by developing their own internal operational guidelines in line with their interpretation, there was still a lack of uniformity [14]. This may indicate that the oral managers were struggling with the processes of policy implementation [9]. There was lack of common understanding on how to achieve the strategic vision as the five districts had different targets and the managers did not have consistent responses on what the expected targets were. This is of concern given that the variation of the targets was not all based on community needs, but rather on what was practically possible. This phenomenon was confirmed by findings from a study assessing implementation activities of oral hygienists at school sites [3].

Although integrated participation and consensus orientation was a policy aspiration, it was not realised at an operational level as there were no mechanisms put in place to establish and facilitate those relationships at a national and provincial level. Furthermore the district managers lacked the agency of building relationships with stakeholders at a local level [23]. The policy was vague on the rule of law and transparency in terms of budgeting and resource allocation. This resulted in variations in implementation, and coping mechanisms in response to the contextual and resource constraints the oral hygienists were facing. They opted for activities which made their work logistically practical, and districts were allowed to use their discretion in terms of selecting targets and activities without having clear accountability measures [8, 14].

The shortage of personnel highlighted by the hygienists has been supported by the literature [24]. Oral health promotion is reported not to have a dedicated budget, oral hygienists make up only 15% of the oral healthcare workforce in the country and their vacancy rate in the public sector is 52% [2, 24, 25]. These limitations affect the hygienist’s responsiveness to local community needs and their ability to widen their reach of schools for addressing oral health inequities. This is reflected by them not being able to visit their designated programmes on a regular basis [3].

There was no mention of quality assurance or directive to address ways of enhancing effectiveness and efficiency [13]. Furthermore the variations in carrying out the policy resulted in the development of duplicate operational guidelines that were hoped to bring about co-ordinated ways of working [8]. As much as the effort was a noteworthy initiative, there was no evidence from the participants that the internal guidelines were widely adopted. Accountability mechanisms of the policy did not apply to all the levels of the system, and it was command driven in order to ensure that the lower authorities comply with the policy [26].

The aim of the monitoring and evaluation plan was to regularly assess the oral health profile and service delivery data, however there was no evidence that the monitoring and evaluation tools included by policy were utilised. The internal operational documents were also not aligned to the national oral health strategy monitoring and evaluation plan. Hence the reliance on the involvement of academics, which was perceived not to be forthcoming. This hampered the ability of the managers to make informed decisions [16]. It can therefore be suggested that the managers be capacitated in utilising the monitoring and evaluation tools for their local decision making [27].

Considering the gaps identified and the expressed perceptions of the participants, various independent studies offer practical solutions to some of these challenges. For instance; Delaney et al. [13], suggest that policy makers at a national level need to ensure that there is a clear understanding of the strategic vision and how that needs to be achieved. They added that this can be achieved by involving participation of all key stakeholders in policy development, establishing initiatives for joint action or through appropriate capacity building and ongoing support [13]. This will ensure shared and joint enforcement of the rule of law, transparency and accountability [13, 16]. Furthermore, it would improve the overall responsiveness, equity and inclusiveness. In addition, adequate resources to facilitate effective implementation, strengthening of routine information systems and surveillance are paramount to tracking and achieving programme targets [27]. The operational managers should optimise their power to improve programme planning, communication and to harmonise service adaptations in response to perennial challenges, using evidence informed solutions [28].

A limitation of the study was that the sample size was small but it included all the key stakeholders responsible for school oral health policy decisions in the province. In addition, there may have been a response bias from the participants which may have distorted their view on the policy. Hence, policy documents were assessed in order to strengthen our study data and verify the information provided by the participants. We cannot claim that the findings are nationally representative as we only interviewed the oral health managers in our target province, however, the results provide baseline information and methodological approach for further research.

## Conclusions

The governance principles adopted from Siddiqi et al. [16] assisted in highlighting the policy implementation challenges experienced by the oral health managers in Gauteng. The policy articulated the governance principles of strategic vision, responsiveness to health needs, equity and inclusivity well, however there were gaps concerning the rest of the governance principles. The principles that were clearly laid out by the policy were not fully and consistently understood or translated by the managers at a local level. As much as the managers attempted to develop local procedural guidelines to ease policy execution, their attempts lacked technical leadership support from the national office and academia in the province.

It is therefore recommended that robust repeated training on policy content is required across all government levels and officials. Furthermore, the training should be tailored to the variety of audiences in order to strengthen a shared understanding of policy and its goals. There is a need for formal structures, such as planning committees or civil society forum that offers policy implementation support, oversight and monitoring at local level.

## Data Availability

Anonymised transcripts will be made available upon reasonable request. The request should be sent to Dr. Mpho Molete, mpho.molete@wits.ac.za.

## Abbreviations

DMFT: Decayed Missing Filled Score
GDoH: Gauteng Provincial Department of Health
NDoH: National Department of Health
